# The Investment (?) Value of Life Insurance

**Published:** 1917-07

**Authors:** 


### BUFFALO MEDICAL JOURNAL

**A Monthly Review of Medicine and Surgery**

EDITOR AND PUBLISHER, DR. A. L. BENEDICT, 228 Summer St., corner of Elmwood Ave., Buffalo, (Address for all communications. Please make personal and telephone calls before 1 P. M.)

Third, in age, on the western continent. Independent, but supports professional organizations. News limited mainly to Western N. Y. and Penn.

Subscription $2.00 a year; $3.00 for two years in advance. Changes and errors in address or failure or duplication of delivery, should be reported immediately.

Yearly Volume 72 JULY, 1917 Number 12

### The Investment (?) Value of Life Insurance.

As agents commonly give the assurance that various forms of policy are good investments, aside from the actual insurance of dependents, we submit the following experience with a 20-year endowment policy in a standard "old line" company, reduced to the basis of $1,000 death benefit:

| | |
|---|---|
| Total premiums paid | $600.01 |
| Total surrender value at end of term | 626.53 |
| Financial profit | 26.52 |
| Value of premiums at 4% simple interest | 849.12 |
| Value of premiums at 4% compounded semi-annually | 929.58 |
| Loss as compared with bank interest as above | 303.05 |

The actuary's statistics showed that an average of a thousand policy holders of equal age, would have to raise from subscribers annually, to pay death claims of $1,000 each, from $4.42 to 10.11. These would amount in 20 years to $206.38. The payment to the company for expenses and profits amounted, in addition, to $96.67. It is unnecessary to state that investments at 5 or 6%, reinvesting interest and payments of principal so as to secure some degree of compounding of interest, pay a great deal more than investment insurance policies. The latter should, therefore, be taken solely as insurance and not with any idea of investment.

Another example is that of a company who offered a term insurance under the name of a 5% gold bond. When the offer was made to buy the bond outright, as an investment, instead of paying annual or semi-annual premiums, it was found that the sum demanded would bear only a fraction over 4% interest.

